# Stupidities

**Published:** 1866-08

**Authors:** 


### STUPIDITIES.

WALKING along the streets with the point of an umbrella sticking out behind, under the arm, or over the shoulder. By suddenly stopping to speak to a friend, or other cause, a person walking in the rear had his brain penetrated through the eye, in one of our streets, and died in a few days.

Stepping into a church aisle, after dismission, and standing to converse with others, or to allow occupants of the same pew to pass out and before, for the courtesy of precedence, at the expense of a greater boorishness to those behind.

To carry a long pencil in vest or outside coat-pocket; not long since, a clerk in New York fell, and the long cedar pencil so pierced an important artery, that it had to be cut down upon from the top of the shoulder, to prevent his bleeding to death, with a three-months' illness.

To take exercise or walk for the health, when every step is a drag, and instinct urges to repose.

To guzzle down glass after glass of cold water, on getting up in the morning, without any feeling of thirst, under the impression of the health-giving nature of its washing-out qualities.

To sit down to a table and "force" yourself to eat when there is not only no appetite, but a positive aversion to food.

To take a glass of soda, or toddy, or sangaree, or mint drops, on a summer day, under the belief that it is safer and better than a glass of cold water.

To economize time, by robbing yourself of necessary sleep, on the ground that an hour saved from sleep is an hour gained for life, when in reality it is two hours actually lost, and half a dozen other hours actually spoiled.

To persuade yourself that you are destroying one unpleasant odor by introducing a stronger one, that is, attempting to sweeten your own unwashed garments and person, by enveloping yourself in the fumes of musk, eau de cologne, or rose-water: the best perfume being, a clean skin and well-washed clothing.

